# Finding Gene Regulatory Networks in Psoriasis: Application of a Tree-Based Machine Learning Approach

**DOI:** 10.3389/fimmu.2022.921408

**Published:** 2022-07-07

**Authors:** Jingwen Deng, Carlotta Schieler, José A. M. Borghans, Chuanjian Lu, Aridaman Pandit

**Affiliations:** ^1^ Center for Translational Immunology, University Medical Center Utrecht, Utrecht University, Utrecht, Netherlands; ^2^ The Second Clinical Medical College, Guangzhou University of Chinese Medicine, Guangzhou, China

**Keywords:** psoriasis, gene regulatory network, machine learning, transcriptome, regulators

## Abstract

Psoriasis is a chronic inflammatory skin disorder. Although it has been studied extensively, the molecular mechanisms driving the disease remain unclear. In this study, we utilized a tree-based machine learning approach to explore the gene regulatory networks underlying psoriasis. We then validated the regulators and their networks in an independent cohort. We identified some key regulators of psoriasis, which are candidates to serve as potential drug targets and disease severity biomarkers. According to the gene regulatory network that we identified, we suggest that interferon signaling represents a key pathway of psoriatic inflammation.

## Introduction

Psoriasis is one of most common chronic inflammatory disorders of the skin, with an estimated 2-3% of adults affected worldwide (1 Psoriasis is characterized by altered keratinocyte differentiation, hyperproliferative epidermis and infiltrating inflammatory cells leading to extensive inflammation (2). Several studies have been performed to understand the molecular pathways underlying psoriasis. The IL-23/IL-17-axis is the most consistent molecular signature underlying psoriasis, resulting in activation of infiltrating immune cells such as myeloid or conventional dendritic cells (mDC or cDC), neutrophils and T-cells, leading to proliferation of keratinocytes (3). However, the exact mechanisms underlying psoriasis remain unclear (4).

One possibility to reveal those underlying mechanisms is based on gene expression data. Using differential gene expression analysis, common differentially expressed genes (DEGs) in psoriasis have been annotated (5). The latter large-scale study also has created a co-expression network of lesional and non-lesional skin, and integrated knowledge about factors such as druggability, genetic association and cell line-specific expression profiles. This analysis however is lacking an inference of regulators in psoriasis. To create such a regulator-target network, different computational approaches have been used. One reliable approach is based on a random forest algorithm, which splits the gene expression data in different learning samples and then finds the networks that most accurately predict the underlying data. The recently developed computational tool RegEnrich also supplies a list of regulators and has shown high accuracy in finding underlying gene regulatory networks (6).

**Figure 1:**
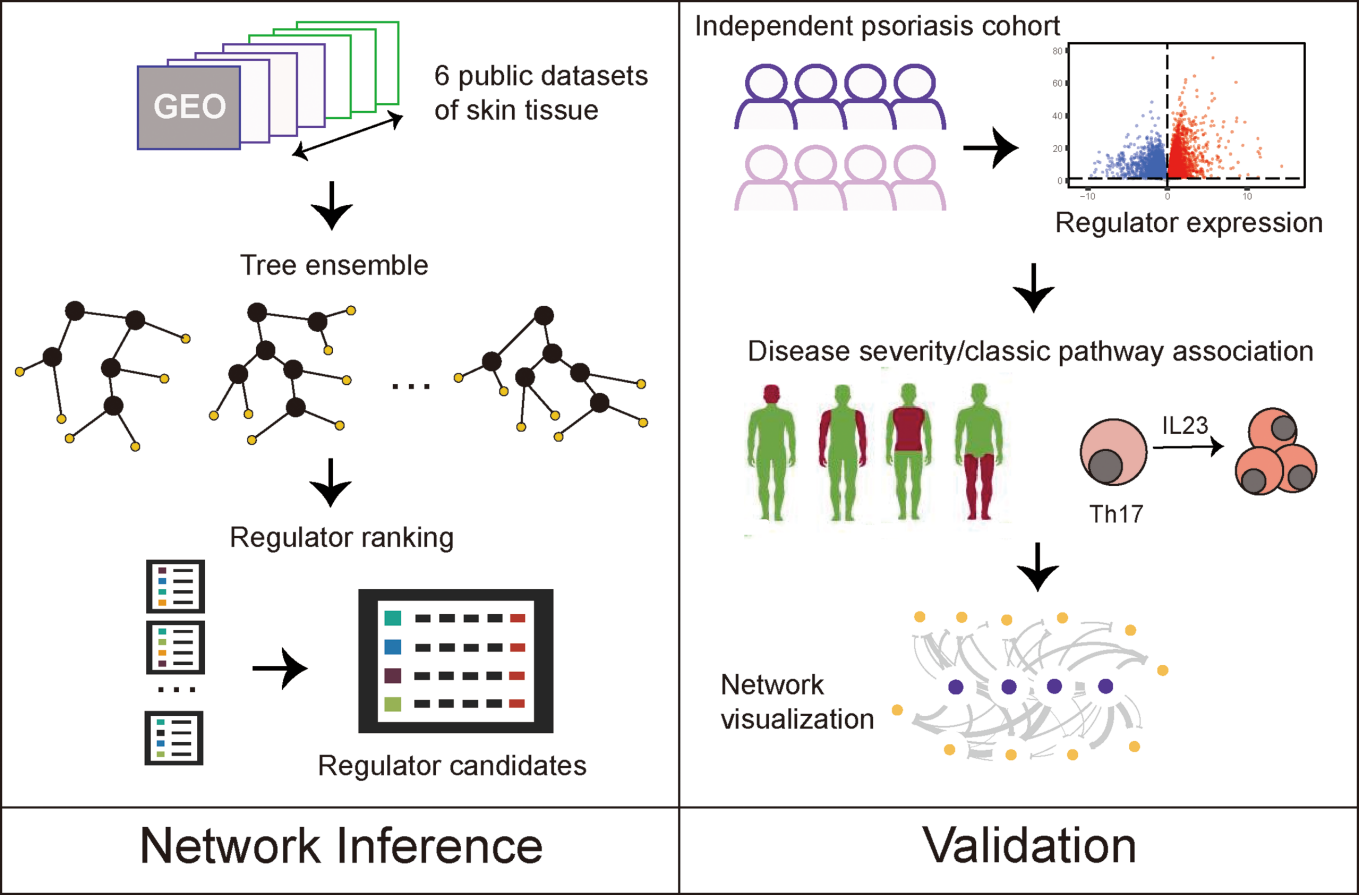
Study designs.

Here, we analyzed large-scale public data to unravel the robust gene signatures in psoriasis using differential gene expression analysis and gene regulatory network analysis of six public cohorts and one independent validation cohort. We compared our results from individuals with psoriasis with those from individuals with atopic dermatitis (AD), to distinguish between specific regulators of psoriatic skin lesions and regulators involved in general skin inflammation (**Figure 1**). We found that psoriasis and AD share many DEGs and pathways, but also identified regulators, such as FOXE1, FOXM1, STAT3 and SOX7, and discovered a gene regulatory network unique for psoriasis.

## Materials and Methods

### Data Acquisition

Four RNA-seq datasets on psoriasis and three on atopic dermatitis were included in this study (**Supplementary Table 1**) (7–11). Among these datasets, three datasets on psoriasis (GSE67785, GSE83645, GSE63979) and one on atopic dermatitis (GSE65832) were obtained from the manually curated and publicly available database by Federico et al. (12). These datasets were already pre-processed, harmonized and prepared according to the FAIR principles (13 The additional RNA-seq datasets GSE121212 (for both psoriasis and AD cohorts) and GSE140227 were retrieved from the gene expression omnibus (GEO) database. Genes with an average count lower of 1 were excluded from all datasets.

### Validation Cohort

As a validation cohort, we used an independent dataset from our previous study (14). This included i) individuals with psoriasis with a dermatologist-confirmed diagnosis of psoriasis, in whom concomitant psoriatic arthritis (PsA) was clinically excluded by a rheumatologist, and ii) individuals with psoriasis and concomitant PsA [fulfilled classification of psoriatic arthritis criteria (CASPAR)] (15). Individuals with a clinical diagnosis of ankylosing spondylitis (AS) [fulfilled assessment of spondyloarthritis international society criteria (ASAS)] (16) were included as a non-psoriatic, inflammatory disease reference group, none of whom had a history of psoriasis (**Supplementary Table 2**). Ethical approval was obtained from the institutional review board before the recruitment of participants. All participants signed written informed consent before participation. As part of a larger overall study design, we performed transcriptome analysis as previously published (14): Briefly, alignment workflow is performed using a two-pass method with STAR (version 020201) (17) to align reads to the reference genome GRCh38 (build 99). The mRNA quantification analysis pipeline measures gene level expression in HT-Seq (version 0.9.0) (18) for raw read count. In differential expression analysis, likelihood ratio test (LRT) was applied for pair-wise comparison between lesion and non-lesion.

### Evaluation of Psoriasis Severity at the Site of Biopsy

For the validation cohort, the psoriasis area severity index (PASI) was calculated by adding up the scores for redness, scaling and thickness of head, arms, trunk and legs and multiplying by the coverage of the plaques (19).

The psoriasis severity index (PSI) was calculated to give a score for the local site where the biopsy/swab of the most-representative affected skin was performed [cumulative score of 0-12 based on the total sum: (0~4 redness) + (0~4 thickness) + (0~4 scaling)] (20).

### Differential Expression Analysis

To find genes that were significantly differentially expressed between lesional and non-lesional skin samples, DEA was carried out using the two-group comparison method Wald significance test of the DESeq2 R-package comparing lesional versus non-lesional samples (version 1.34.0) (21). To make sure that all setting were the same for all public datasets, design formula was simply set as “design =~lesional” and “coef=lesional_lesional_vs_non_lesional”. To identify the biological function of the DEGs, pathway enrichment analysis was carried out using the function gseGO, enrichKEGG and enrichPathway of the R package clusterProfiler (version 4.2.0) (22).

### Gene Regulatory Network Analysis

The results from DEA were used to infer a regulator-target network using a random forest and gene set enrichment analysis (GSEA) approach implemented in the RegEnrich R package (version 1.4.0), with the parameters: method = “Wald_DESeq2, networkConstruction = “GRN”, enrichTest = “GSEA”, and default transcription factor list (6). Using RegEnrich, we analyzed the regulator-target relationships between transcription factors and the DEGs obtained from the psoriatic lesional and non-lesional comparison, allowing us to rank the regulators based on their importance in each of the studied cohorts. We selected the regulators that were in the front median rank across all datasets and which correlated with the disease severity and biomarkers of psoriasis, and then visualized the regulatory network using Cytoscape (version 3.8.0) (23). Between Lane Normalization (BLN) and Z-score transformation were used for the heatmap visualization of the gene expression of regulatory network with pheatmap R package (version 1.0.12).

### Statistical Analysis

Spearman rank correlations were calculated between gene expression and clinical parameters with correlation R package (version 0.8.0). For differential expression analysis, P values were automatically adjusted with the Benjamini-Hochberg (BH) method with the function DESeq; For the correlation analysis, BH adjustment was applied with function correlation and parameter “p_adjust = ‘BH’”; For the hypergeometric tests in pathway enrichment analysis, BH adjustment was performed with parameter “pAdjustMethod = ‘BH’” in function gseGO, enrichKEGG and enrichPathway. For pathway enrichment analysis, adjusted *P* value of  0.2 was used as the cutoff for significance. For other tests, adjusted *P* values < 0.05 were considered statistically significant. All statistical analyses were performed using R (version 4.0.3) (http://cran.r-project.org/).

## Results

### Psoriasis Shares Most of the Immune-Related Pathways With AD

**Figure 2:**
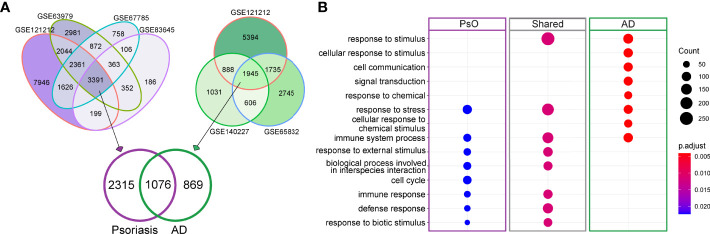
DEGs for psoriasis and AD and GO enrichment analysis of shared and disease-specific DEGs. **(A)** Venn diagram visualizing the overlaps in DEGs between lesional and non-lesional skin in different public datasets for psoriasis (purple) and AD (green). **(B)** Involved GO pathways based on the gene lists of psoriasis-specific DEGs, AD-specific DEGs and DEGs that are shared between psoriasis and AD. The size of dot denotes number of DEGs involved in the term, while color denotes the adjusted *P* value of the hypergeometric test for gene enrichment.

We first analyzed the DEGs between lesional and non-lesional skin across all included public datasets (four datasets for psoriasis and three for AD, **Supplementary Table 1**). Wesix first looked for similarities between the datasets for each disease, and found a considerable number of overlapping DEGs, i.e., 3391 and 1945 consensus genes for psoriasis and AD, respectively. We then studied the overlap between these consensus genes in psoriasis and AD and found 1076 genes to be shared by these two diseases (**Figure 2A**, **Supplementary Table 4**). This finding suggests a large similarity in gene expression profile between psoriasis and AD. We used these sets of commonly expressed genes and disease-specific genes to carry out a gene ontology (GO) enrichment analysis to find their functional similarities and differences. As expected, the shared genes display some common inflammatory pathways, mainly the activation of immune system pathways (**Figure 2B**). The differential expression of genes in the cell cycle pathway is unique for psoriasis, and may relate to epithelial hyperplasia and hyper-keratinization. The dominance of IL-13 pathways in AD is presented in Reactome pathway enrichment, which is consistent with the recent study (11 In summary, psoriasis has discernable auto-immunological activations, while AD has increased presence of dermal response to stimulus.

### Unique Regulators Highlight Differences of Psoriasis and AD

**Figure 3:**
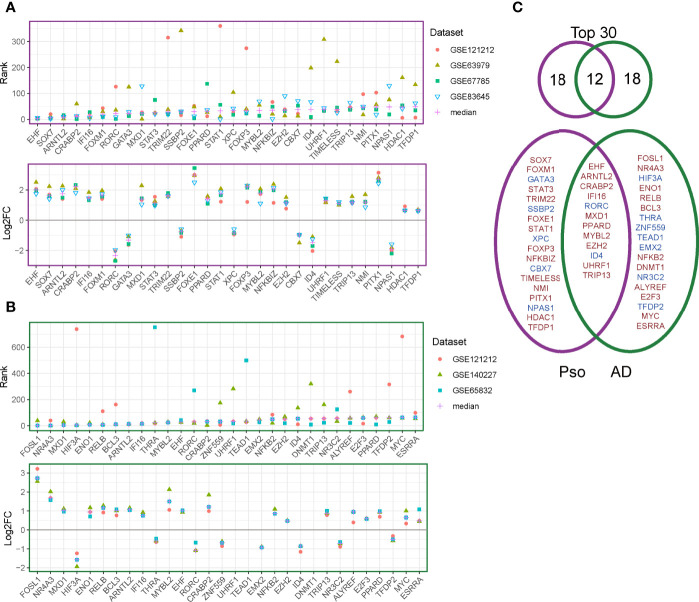
Regulator discovery on publicly available datasets. **(A)** The ranks (upper panel) and log2 fold change (between lesional and non-lesional skin) (lower panel) of the top 30 regulators in the psoriasis datasets. **(B)** The ranks (upper panel) and log2 fold change (between lesional and non-lesional skin) (lower panel) of the top 30 regulators in the AD datasets. **(C)** Venn diagram for the overlapping and disease-specific regulators in psoriasis and AD. Regulators given in red denote up-regulated genes and those in blue denote down-regulated regulators.

We further explored the public datasets to identify gene regulators consistent across multiple psoriasis and AD datasets. We used a random forest algorithm (as part of the RegEnrich package) to construct the gene-regulatory networks of each individual dataset. Using RegEnrich, we identified the top 30 regulators for each disease that were consistently ranking as the top regulators across multiple datasets corresponding to each disease (**Figure 3A**). We found several regulators that have previously been reported to be increased in psoriasis, such as CRABP2 (a carrier protein for retinoic acid signaling pathways) and STAT3 (a central regulator of various immune responses) (24–26) (**Figure 3B**). We also identified some regulators that were decreased in the psoriatic condition. For instance, GATA3, a transcription factor for the differentiation of Th2 cells, which is typically increased in AD (27), was down-regulated in psoriatic lesions. Among the top 30 regulators, we identified 18 disease-specific regulators for each disease; the remaining 12 regulators were shared between psoriasis and AD and may thus be involved in general inflammation of the skin (**Figure 3C**).

### Independent Cohort to Validate Regulators of Psoriasis

**Figure 4:**
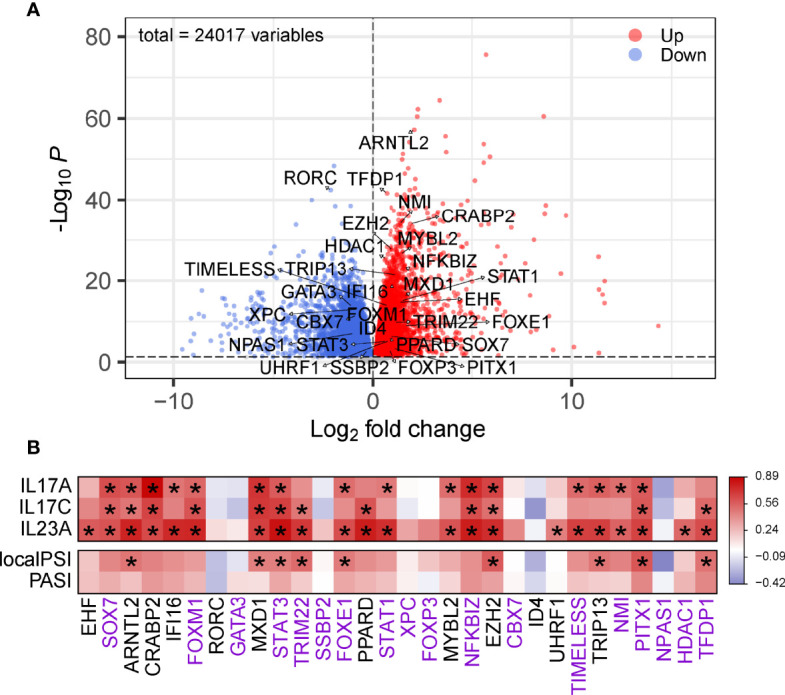
Validation of the identified regulators. **(A)** Volcano plot of DEGs in psoriatic lesional compared to non-lesional skin. The top 30 regulators are labeled with their names. The red dots are the genes with both a false discovery rate (FDR) smaller than 0.05 and a log2FoldChange greater than 0. The blue dots are the genes with either an FDR smaller than 0.05 or a log2FoldChange smaller than 0. **(B)** Heatmap of correlation between the top 30 regulators and gene markers or measures of disease severity. Color denotes *r* value of correlation. The gene names with purple are considered as unique DEGs of psoriasis, with black are shared genes with AD. * Adjusted *P* < 0.05.

**Figure 5:**
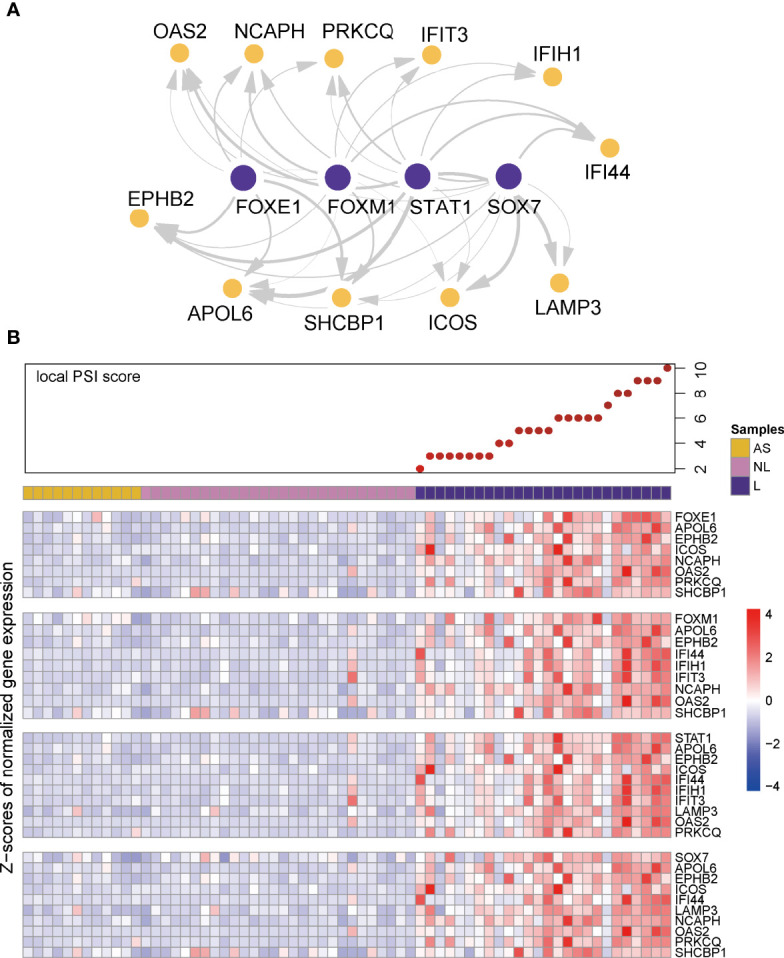
Visualization of gene regulatory network and co-expression pattern. **(A)** Visualization of gene regulatory network in psoriasis. Edge width represents the weight of each connection. **(B)** Local PSI score and heatmaps of co-expression pattern of key regulators (first row of each heatmap panel) and their target genes across all the samples in the validation cohort.

To validate the identified top regulators and to connect them to clinical parameters of psoriasis, we used one of our own recently produced datasets comprising transcriptome profiles of 40 skin samples from a cohort of individuals with psoriasis, psoriatic arthritis or ankylosing spondylitis. All the top 30 regulators were differentially expressed between lesion and non-lesion samples (**Figure 4A**, **Supplementary Table 4**) (14). We further analyzed the correlation between the expression of these regulators and some well-known gene biomarkers of psoriasis (i.e. IL17A, IL17C, IL23A) and measures of disease severity (i.e. the PASI and PSI). Remarkably, 22 out of the 30 identified regulators correlated significantly with at least one of the biomarkers of psoriasis in our cohort, showing the robustness of our method. Nine regulators were significantly positively correlated with disease severity (**Figure 4B**). Of these nine regulators, four regulators (STAT3, FOXE1,PITX1,TFD) did not overlap with the top 30 regulators of AD, we consider these four regulators as key regulators for psoriasis. Thus, we visualized the gene regulatory network for psoriasis with these key regulators and their target genes, based on the representation of regulator-target connectivity across all discovery sets and the validation set (**Figure 5A**). We found that these regulators and their target genes were highly expressed in lesions, compared to non-lesions or skin from individuals with AS. The regulators and their target genes showed a coordinated expression pattern across all samples (**Figure 5B**).

In summary, we validated the gene regulatory networks in our independent cohort, in terms of differential gene expression, clinical phenotype association, and regulator-target gene co-expression.

## Discussion

Psoriasis is one of many inflammatory skin diseases. Here, we used a machine learning approach to reveal the underlying gene regulatory networks and to identify the gene regulators responsible for the activity of several other genes. To evaluate if the regulators that we identified are specific for psoriasis or are commonly found in inflammatory skin, we repeated the gene regulatory network inference with datasets of AD. Comparing our results of both diseases, we found a large overlap in the involved pathways. Nevertheless, we were able to highlight distinct regulators for psoriasis and AD. These regulators might help further studies to understand both diseases in more detail.

Our approach firstly generated a list of common and specific DEGs of psoriasis and AD for further investigation. 3391 and 1945 consensus DEGs for psoriasis and AD, respectively. Among these consensus DEGs, 1076 DEGs are shared by these two diseases. Many of the consensus DEGs are known to be relevant to psoriasis or AD. For example, IL17 families (IL17A, IL17C, IL17D, IL17F, IL17RC, IL17RD, IL17RE) were consistently differentially expressed in all psoriatic datasets but not in AD. By contrast, IL-6 that mainly produced by Th2 cells, is a specific DEG for AD. Interestingly, keratinocyte is involved in the pathogenetic of both psoriasis and AD, but the profile of keratins is distinct between these two diseases. KRT15, KRT24, KRT31, KRT37, KRT78 are differentially expressed in psoriasis, while KRT5 and KRT14 are specific for AD. Further psoriasis and AD share the KRT6A, KRT6B, KRT6C, KRT16, KRT17 as their common DEGs. This pattern provides us more information about the role keratins play in modulation of the immune response in skin. In the pathway analysis, we found that most of the dysregulated pathways in psoriasis being shared with AD. This finding has been reported in some comparative transcriptomic studies of psoriasis and AD (11, 28

Further, we identified the gene regulators in psoriasis and AD. After the regulator ranking, we utilized our independent cohort to validate the top 30 regulators of psoriasis. Most of these regulators were associated with the expression of IL17 and IL23. Four regulators were correlated with disease severity and are therefore considered to be key regulators of psoriasis. STAT3 plays an important role in inflammatory responses, and is implicated in the pathogenesis of psoriasis. It has been reported to mediate a majority of cytokines in the development of psoriasis, including the central IL-23/IL-17 axis (24, 29, 30, p. 3; 31, 32). The discovery of the central role of STAT3 signaling in psoriasis pathogenesis has promoted the development of new drugs such as JAK inhibitors (33). FOXE1 is a member of the forkhead box family. A previous study found FOXE1 to be upregulated after IL-17 or TNF simulation on keratinocytes (34). Notably, two additional forkhead box family members (FOXM1 and FOXP3) are also ranked within the top 30 regulators in psoriasis. This might indicate the involvement of this family in the development of psoriasis. TFDP1 is a member of E2F family that control the transcriptional activity of numerous genes involved in cell cycle progression from G1 to S phase. It was identified as central node with another systems biology approach, suggesting it might play a role in the keratinocyte hyperproliferation in psoriasis (35). PITX1 is a member of the RIEG/PITX homeobox family which is involved in organ development and is yet not associated with psoriasis.

We noticed that the four identified key regulators shared most of their target genes with each other. Some of these target genes have been reported as psoriasis-associated genes. For instance, PRKCQ and EPHB2 are reported to be overexpressed in psoriatic skin (36, 37). Interestingly, interferon-induced gene families (IFIT3 and IFI16) are involved in this gene regulatory network, which suggests that inflammation is affected by these regulators. APOL6 is indicated to be mediated by interferon-γ in atherosclerotic cells (38). SHCBP1 is one of the host proteins interacting with the C protein which counteracts the host interferon response (39). These target genes are all involved in the interferon signaling pathway (40). This is of particular importance since interferon signaling represents a key feature of psoriatic inflammation. Previous studies demonstrated the interferon responses, especially IFITM1, IFITM2, and CCL5 were increased of in skin of patients with AD (41, 42). In our study, we found a prioritized gene regulatory network of interferon signaling in psoriasis but not in AD, suggesting the interferon-mediated priming can contribute to the responses to different inflammatory factors in different conditions. In conclusion, the combination of differential gene expression, tree-based gene regulatory network inference and enrichment analysis on large-scale datasets reveals a set of key regulators in both psoriasis and AD. The identified genes, pathways, and regulators are consistent with the current knowledge about psoriasis and AD, reflecting the credibility of our applied methods. Moreover, we identified some key regulators of psoriasis, which are candidates to serve as disease severity biomarkers and potential drug targets.

However, our study only includes a relatively small number of public datasets. With increasing access to publicly available psoriasis and AD datasets, our approach serves a robust way to integrate large amount of data. Also, we found that the regulators ranked after top 30 were less associated with IL23/IL17 and disease severity (**Supplementary Figure 3**), so that we only included top 30 regulators for the validation and network visualization, which do not cover all potential regulators of interest. Furthermore, we could validate the network with our psoriatic cohort, but may not have been able to validate our findings with inhouse AD cohort. To assess the clinical relevance of these identified regulators, further analytical and laboratory studies will be necessary.

## Data Availability Statement

The skin transcriptome data for validation is available in the GEO database (GSE186063).

## Ethics Statement

The studies involving human participants were reviewed and approved by The Institutional Review Board of University Medical Centre Utrecht. The patients/participants provided their written informed consent to participate in this study.

## Author Contributions

Conceptualization: AP and JD; Methodology: AP, JB, JD, and CS; Data curation: JD and CS; Formal analysis: JD and CS; Project administration: AP, JB, and CL; Supervision: AP, JB, and CL; Resources: JD and CS; Writing – original draft: CS, JD, AP, JB, and CL; Writing – review and editing: JD, CS, AP, JB, and CL. All authors contributed to the article and approved the submitted version.

## Funding

JD was supported by the China Scholarship Council (CSC) NO. 202007720051; National Natural Science Foundation of China (U20A20397) and Science and Technology Planning Project of Guangdong Province (2020B1111100005).

## Conflict of Interest

The authors declare that the research was conducted in the absence of any commercial or financial relationships that could be construed as a potential conflict of interest.

## Publisher’s Note

All claims expressed in this article are solely those of the authors and do not necessarily represent those of their affiliated organizations, or those of the publisher, the editors and the reviewers. Any product that may be evaluated in this article, or claim that may be made by its manufacturer, is not guaranteed or endorsed by the publisher.

## Supplementary Material

Supplementary Figure 1 Reactome Pathway and KEGG annotation for DEGs. **(A)** Involved Reactome pathways based on the gene lists of psoriasis-specific DEGs, AD-specific DEGs and DEGs that are shared between psoriasis and AD. The size of dot denotes number of DEGs involved in the term, while color denotes the adjusted *P* value of the hypergeometric test for gene enrichment. **(B)** Involved KEGG pathways based on the gene lists of psoriasis-specific DEGs, AD-specific DEGs and DEGs that are shared between psoriasis and AD. The size of dot denotes number of DEGs involved in the term, while color denotes the adjusted *P* value of the hypergeometric test for gene enrichment. **(B)** Involved KEGG pathways based on the top 500 genes of psoriasis-specific DEGs, top 500 genes of AD-specific DEGs and top 500 genes of DEGs that are shared between psoriasis and AD. The size of dot denotes number of DEGs involved in the term, while color denotes the adjusted *P* value of the hypergeometric test for gene enrichment.

Supplementary Figure 2 Venn diagram for the overlapping and disease-specific regulators in psoriasis and AD, at different levels. Regulators given in red denote up-regulated genes and those in blue denote down-regulated regulators.

Supplementary Figure 3 Heatmap of correlation between the top 50 regulators and gene markers or measures of disease severity. Color denotes *r* value of correlation. The gene names with purple are considered as unique DEGs of psoriasis, with black are shared genes with AD. * Adjusted *P* < 0.05

## References

[1] Parisi R Symmons DPM Griffiths CEM Ashcroft DM & Identification and Management of Psoriasis and Associated ComorbidiTy (IMPACT) project team. Global Epidemiology of Psoriasis: A Systematic Review of Incidence and Prevalence. J Invest Dermatol (2013) 133(2):377–85. doi: 10.1038/jid.2012.339 23014338

[2] Albanesi C Madonna S Gisondi P Girolomoni G. The Interplay Between Keratinocytes and Immune Cells in the Pathogenesis of Psoriasis. Front Immunol (2018) 9:1549. doi: 10.3389/fimmu.2018.01549 30034395 PMC6043636

[3] Fotiadou C Lazaridou E Sotiriou E Gerou S Kyrgidis A Vakirlis E. IL-17a, IL-22, and IL-23 as Markers of Psoriasis Activity: A Cross-Sectional, Hospital-Based Study. J Cutaneous Med Surg (2015) 19(6):555–60. doi: 10.1177/1203475415584503 25917082

[4] Ayala-Fontánez N Soler DC McCormick TS. Current Knowledge on Psoriasis and Autoimmune Diseases. Psoriasis (Auckland NZ) (2016) 6:7–32. doi: 10.2147/PTT.S64950 PMC5683130 29387591

[5] Zeng F Liu H Lu D Liu Q Chen H Zheng F. Integrated Analysis of Gene Expression Profiles Identifies Transcription Factors Potentially Involved in Psoriasis Pathogenesis. J Cell Biochem (2019) 120(8):12582–94. doi: 10.1002/jcb.28525 30825251

[6] Tao W Radstake TRDJ Pandit A. RegEnrich Gene Regulator Enrichment Analysis Reveals a Key Role of the ETS Transcription Factor Family in Interferon Signaling. Commun Biol (2022) 5:31. doi: 10.1038/s42003-021-02991-5 35017649 PMC8752721

[7] Suárez-Fariñas M Ungar B Rosa JC Ewald DA Rozenblit M Gonzalez J. RNA Sequencing Atopic Dermatitis Transcriptome Profiling Provides Insights Into Novel Disease Mechanisms With Potential Therapeutic Implications. J Allergy Clin Immunol (2015) 135(5):1218–27. doi: 10.1016/j.jaci.2015.03.003 25840722

[8] Swindell WR Remmer HA Sarkar MK Xing X Barnes DH Wolterink L. Proteogenomic Analysis of Psoriasis Reveals Discordant and Concordant Changes in mRNA and Protein Abundance. Genome Med (2015) 7(1):86. doi: 10.1186/s13073-015-0208-5 26251673 PMC4527112

[9] Tsoi LC Iyer MK Stuart PE Swindell WR Gudjonsson JE Tejasvi T. Analysis of Long Non-Coding RNAs Highlights Tissue-Specific Expression Patterns and Epigenetic Profiles in Normal and Psoriatic Skin. Genome Biol (2015) 16:24. doi: 10.1186/s13059-014-0570-4 25723451 PMC4311508

[10] Tsoi LC Yang J Liang Y Sarkar MK Xing X Beamer MA. Transcriptional Determinants of Individualized Inflammatory Responses at Anatomically Separate Sites. J Allergy Clin Immunol (2018) 141(2):805–8. doi: 10.1016/j.jaci.2017.07.054 PMC5861732 29031600

[11] Tsoi LC Rodriguez E Degenhardt F Baurecht H Wehkamp U Volks N. Atopic Dermatitis Is an IL-13-Dominant Disease With Greater Molecular Heterogeneity Compared to Psoriasis. J Invest Dermatol (2019) 139(7):1480–9. doi: 10.1016/j.jid.2018.12.018 PMC6711380 30641038

[12] Federico A Hautanen V Christian N Kremer A Serra A Greco D. Manually Curated and Harmonised Transcriptomics Datasets of Psoriasis and Atopic Dermatitis Patients. Sci Data (2020) 7:343. doi: 10.1038/s41597-020-00696-8 33051456 PMC7555498

[13] Wilkinson MD Dumontier M Aalbersberg IJJ Appleton G Axton M Baak A. The FAIR Guiding Principles for Scientific Data Management and Stewardship. Sci Data (2016) 3:160018. doi: 10.1038/sdata.2016.18 26978244 PMC4792175

[14] Deng J Leijten E Nordkamp MO Hartgring S Tao W Pouw J. Interactions of host defense and hyper-keratinization in psoriasis. (2021). p. 2021.11.26.469424. doi: 10.1101/2021.11.26.469424.

[15] Taylor W Gladman D Helliwell P Marchesoni A Mease P Mielants H. Classification Criteria for Psoriatic Arthritis: Development of New Criteria From a Large International Study. Arthritis Rheum (2006) 54(8):2665–73. doi: 10.1002/art.21972 16871531

[16] Rudwaleit M van der Heijde D Landewé R Listing J Akkoc N Brandt J. The Development of Assessment of SpondyloArthritis International Society Classification Criteria for Axial Spondyloarthritis (Part II): Validation and Final Selection. Ann Rheum Dis (2009) 68(6):777–83. doi: 10.1136/ard.2009.108233 19297344

[17] Dobin A Davis CA Schlesinger F Drenkow J Zaleski C Jha S. STAR: Ultrafast Universal RNA-Seq Aligner. Bioinf (Oxford England) (2013) 29(1):15–21. doi: 10.1093/bioinformatics/bts635 PMC3530905 23104886

[18] Anders S Pyl PT Huber W. HTSeq—A Python Framework to Work With High-Throughput Sequencing Data. Bioinf (Oxford England) (2015) 31(2):166–9. doi: 10.1093/bioinformatics/btu638 PMC4287950 25260700

[19] Fredriksson T Pettersson U. Severe Psoriasis—Oral Therapy With a New Retinoid. Dermatologica (1978) 157(4):238–44. doi: 10.1159/000250839 357213

[20] Hoffmann JHO Enk AH. Evaluation of Psoriasis Area and Severity Index Thresholds as Proxies for Systemic Inflammation on an Individual Patient Level. Dermatol (Basel Switzerland) (2021) 1–6. doi: 10.1159/000520163 34852349

[21] Love MI Huber W Anders S. Moderated Estimation of Fold Change and Dispersion for RNA-Seq Data With Deseq2. Genome Biol (2014) 15(12):550. doi: 10.1186/s13059-014-0550-8 25516281 PMC4302049

[22] Wu T Hu E Xu S Chen M Guo P Dai Z. Clusterprofiler 4.0: A Universal Enrichment Tool for Interpreting Omics Data. Innovation (New York NY) (2021) 2(3):100141. doi: 10.1016/j.xinn.2021.100141 PMC8454663 34557778

[23] Smoot ME Ono K Ruscheinski J Wang P-L Ideker T. Cytoscape 2.8: New Features for Data Integration and Network Visualization. Bioinf (Oxford England) (2011) 27(3):431–2. doi: 10.1093/bioinformatics/btq675 PMC3031041 21149340

[24] Calautti E Avalle L Poli V. Psoriasis: A STAT3-Centric View. Int J Mol Sci (2018) 19(1):171. doi: 10.3390/ijms19010171 PMC5796120 29316631

[25] Keermann M Kõks S Reimann E Prans E Abram K Kingo K. Transcriptional Landscape of Psoriasis Identifies the Involvement of IL36 and IL36RN. BMC Genomics (2015) 16(1):322. doi: 10.1186/s12864-015-1508-2 25897967 PMC4405864

[26] Lundberg KC Fritz Y Johnston A Foster AM Baliwag J Gudjonsson JE. Proteomics of Skin Proteins in Psoriasis: From Discovery and Verification in a Mouse Model to Confirmation in Humans. Mol Cell Proteomics: MCP (2015) 14(1):109–19. doi: 10.1074/mcp.M114.042242 PMC4288247 25351201

[27] Barnes PJ. Role of GATA-3 in Allergic Diseases. Curr Mol Med (2008) 8(5):330–4. doi: 10.2174/156652408785160952 18691059

[28] Choy DF Hsu DK Seshasayee D Fung MA Modrusan Z Martin F. Comparative Transcriptomic Analyses of Atopic Dermatitis and Psoriasis Reveal Shared Neutrophilic Inflammation. J Allergy Clin Immunol (2012) 130(6):1335–43.e5. doi: 10.1016/j.jaci.2012.06.044 22920495 PMC3511596

[29] Holland SM DeLeo FR Elloumi HZ Hsu AP Uzel G Brodsky N. STAT3 Mutations in the Hyper-IgE Syndrome. N Engl J Med (2007) 357(16):1608–19. doi: 10.1056/NEJMoa073687 17881745

[30] Ma CS Chew GYJ Simpson N Priyadarshi A Wong M Grimbacher B. Deficiency of Th17 Cells in Hyper IgE Syndrome Due to Mutations in STAT3. J Exp Med (2008) 205(7):1551–7. doi: 10.1084/jem.20080218 PMC2442632 18591410

[31] Milner JD Brenchley JM Laurence A Freeman AF Hill BJ Elias KM. Impaired T(H)17 Cell Differentiation in Subjects With Autosomal Dominant Hyper-IgE Syndrome. Nature (2008) 452(7188):773–6. doi: 10.1038/nature06764 PMC2864108 18337720

[32] Minegishi Y Saito M Tsuchiya S Tsuge I Takada H Hara T. Dominant-Negative Mutations in the DNA-Binding Domain of STAT3 Cause Hyper-IgE Syndrome. Nature (2007) 448(7157):1058–62. doi: 10.1038/nature06096 17676033

[33] Di Lernia V Bardazzi F. Profile of Tofacitinib Citrate and Its Potential in the Treatment of Moderate-to-Severe Chronic Plaque Psoriasis. Drug Design Dev Ther (2016) 10:533–9. doi: 10.2147/DDDT.S82599 PMC4743637 26889081

[34] Chiricozzi A Guttman-Yassky E Suárez-Fariñas M Nograles KE Tian S Cardinale I. Integrative Responses to IL-17 and TNF-α in Human Keratinocytes Account for Key Inflammatory Pathogenic Circuits in Psoriasis. J Invest Dermatol (2011) 131(3):677–87. doi: 10.1038/jid.2010.340 21085185

[35] Manczinger M Kemény L. Novel Factors in the Pathogenesis of Psoriasis and Potential Drug Candidates are Found With Systems Biology Approach. PLoS One (2013) 8(11):e80751. doi: 10.1371/journal.pone.0080751 24303025 PMC3841158

[36] Delić D Wolk K Schmid R Gabrielyan O Christou D Rieber K. Integrated microRNA/mRNA Expression Profiling of the Skin of Psoriasis Patients. J Dermatol Sci (2020) 97(1):9–20. doi: 10.1016/j.jdermsci.2019.11.003 31843230

[37] Li Y Begovich AB. Unraveling the Genetics of Complex Diseases: Susceptibility Genes for Rheumatoid Arthritis and Psoriasis. Semin Immunol (2009) 21(6):318–27. doi: 10.1016/j.smim.2009.04.002 19446472

[38] Zhaorigetu S Yang Z Toma I McCaffrey TA Hu C-AA. Apolipoprotein L6, Induced in Atherosclerotic Lesions, Promotes Apoptosis and Blocks Beclin 1-Dependent Autophagy in Atherosclerotic Cells. J Biol Chem (2011) 286(31):27389–98. doi: 10.1074/jbc.M110.210245 PMC3149332 21646352

[39] Ito M Iwasaki M Takeda M Nakamura T Yanagi Y Ohno S. Measles Virus Nonstructural C Protein Modulates Viral RNA Polymerase Activity by Interacting With Host Protein Shcbp1. J Virol (2013) 87(17):9633–42. doi: 10.1128/JVI.00714-13 PMC3754092 23804634

[40] Lin J Li X Zhang F Zhu L Chen Y. Transcriptome Wide Analysis of Long Non-Coding RNA-Associated ceRNA Regulatory Circuits in Psoriasis. J Cell Mol Med (2021) 25(14):6925–35. doi: 10.1111/jcmm.16703 PMC8278092 34080300

[41] Lefèvre-Utile A Saichi M Oláh P Delord M Homey B Soumelis V. Transcriptome-Based Identification of Novel Endotypes in Adult Atopic Dermatitis. Allergy (2022) 77(5):1486–98. doi: 10.1111/all.15150 34689335

[42] Rebane A Zimmermann M Aab A Baurecht H Koreck A Karelson M. Mechanisms of IFN-γ–Induced Apoptosis of Human Skin Keratinocytes in Patients With Atopic Dermatitis. J Allergy Clin Immunol (2012) 129(5):1297–306. doi: 10.1016/j.jaci.2012.02.020 22445417

