# Supplementary figures and images for: Finding Gene Regulatory Networks in Psoriasis: Application of a Tree-Based Machine Learning Approach
Source: Front Immunol. 2022 Jul 7;13:921408. doi: 10.3389/fimmu.2022.921408 (PMC9301015; doi:10.3389/fimmu.2022.921408)

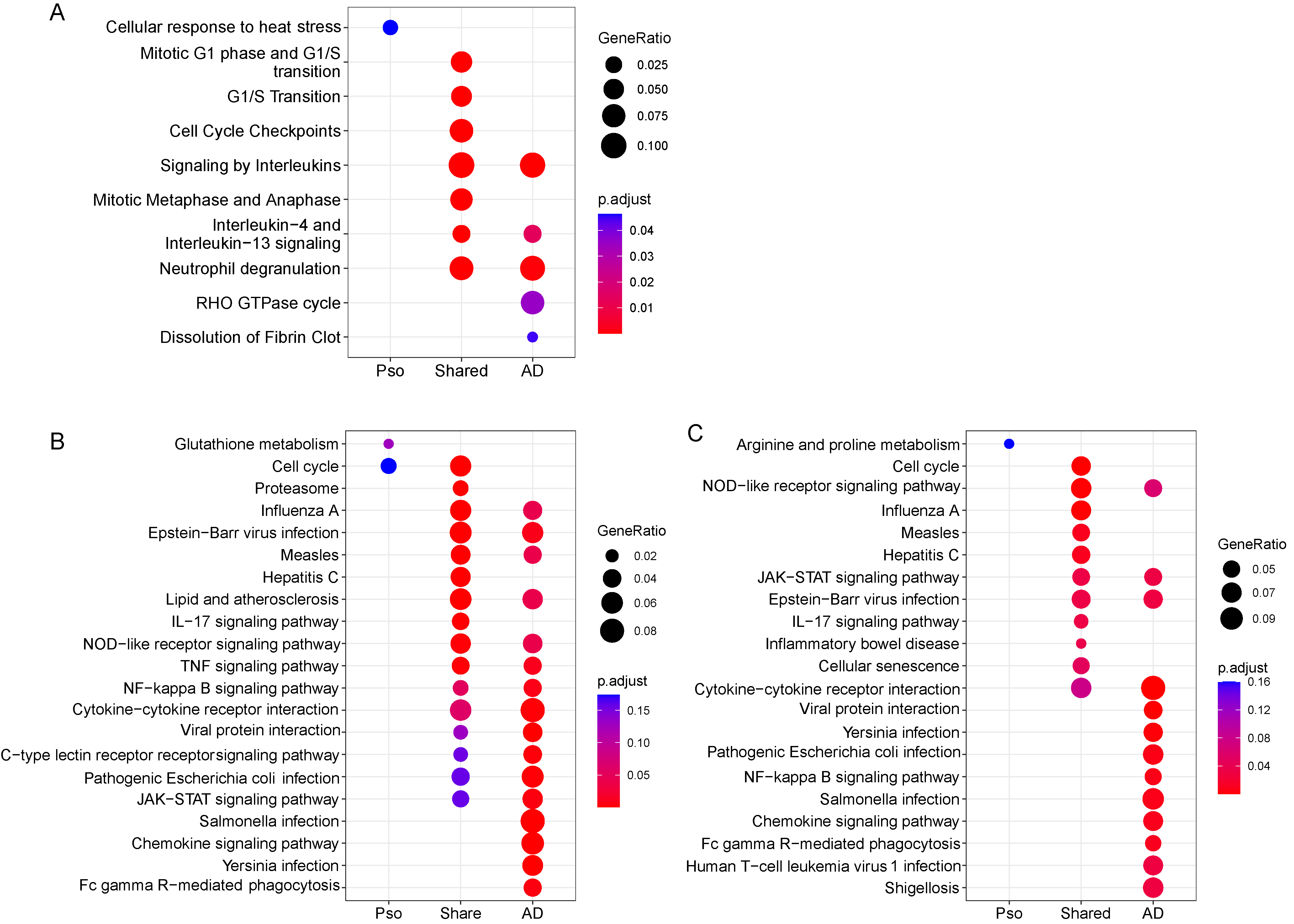

Supplement: Supplementary Figure 1 — Reactome Pathway and KEGG annotation for DEGs. (A) Involved Reactome pathways based on the gene lists of psoriasis-specific DEGs, AD-specific DEGs and DEGs that are shared between psoriasis and AD. The size of dot denotes number of DEGs involved in the term, while color denotes the adjusted P value of the hypergeometric test for gene enrichment. (B) Involved KEGG pathways based on the gene lists of psoriasis-specific DEGs, AD-specific DEGs and DEGs that are shared between psoriasis and AD. The size of dot denotes number of DEGs involved in the term, while color denotes the adjusted P value of the hypergeometric test for gene enrichment. (B) Involved KEGG pathways based on the top 500 genes of psoriasis-specific DEGs, top 500 genes of AD-specific DEGs and top 500 genes of DEGs that are shared between psoriasis and AD. The size of dot denotes number of DEGs involved in the term, while color denotes the adjusted P value of the hypergeometric test for gene enrichment. [file Image_1.tif]

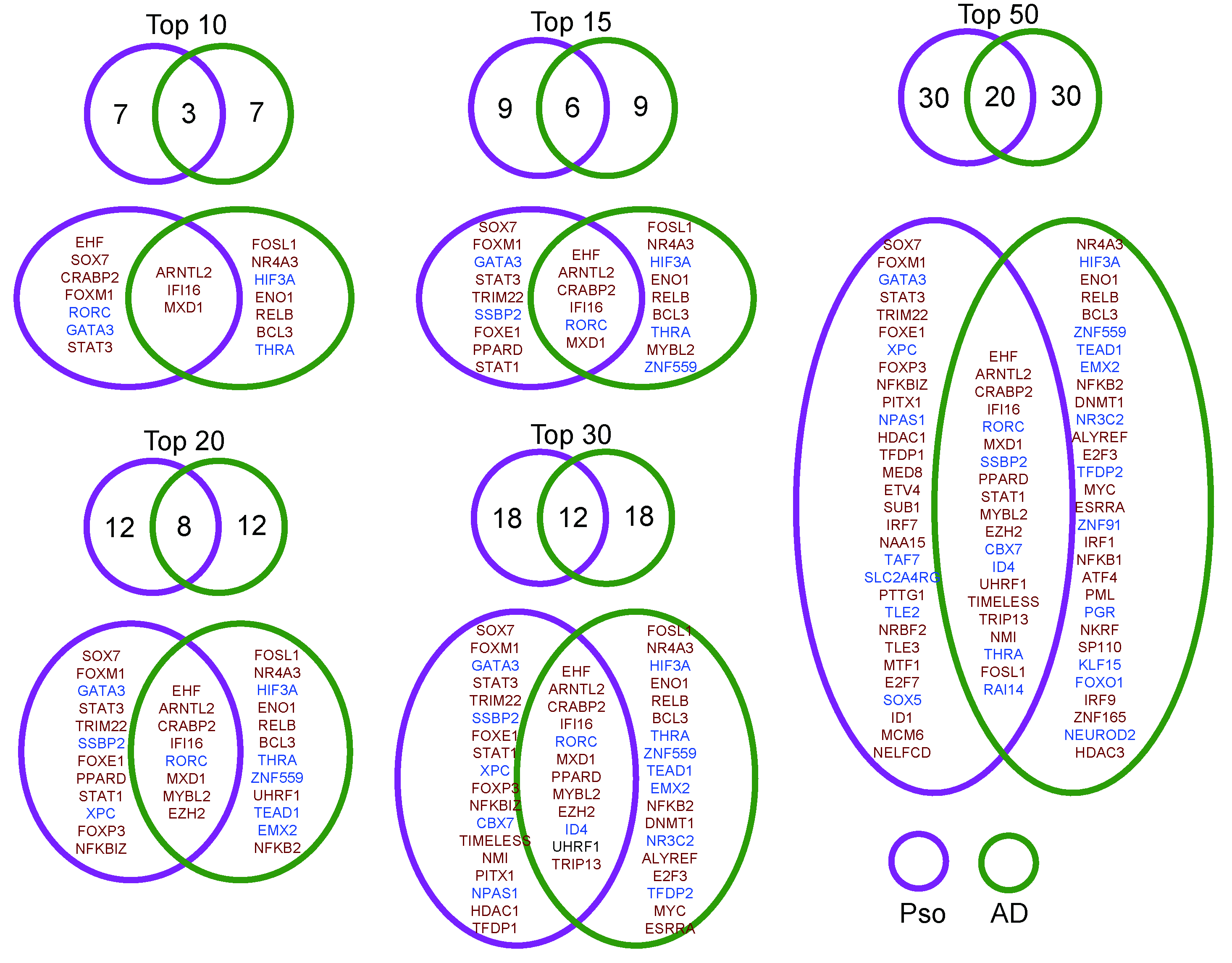

Supplement: Supplementary Figure 2 — Venn diagram for the overlapping and disease-specific regulators in psoriasis and AD, at different levels. Regulators given in red denote up-regulated genes and those in blue denote down-regulated regulators. [file Image_2.tif]

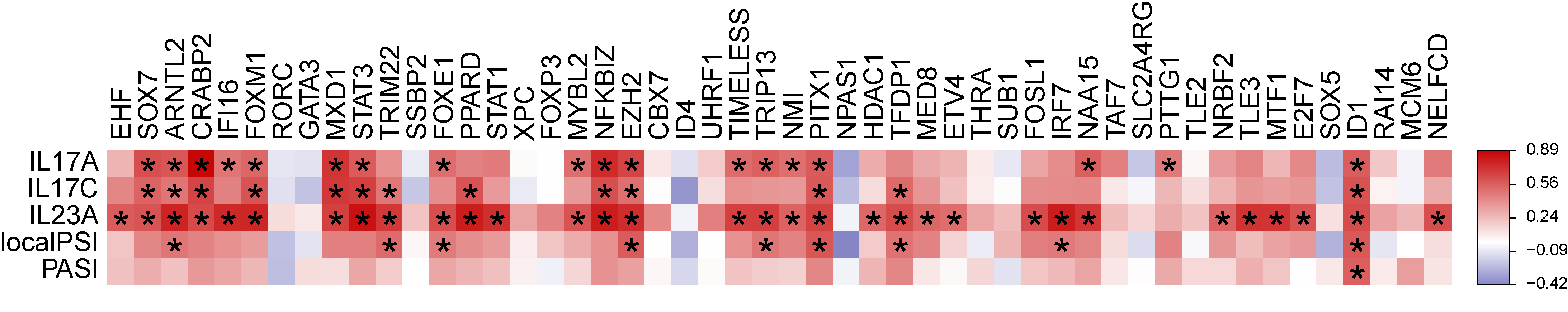

Supplement: Supplementary Figure 3 — Heatmap of correlation between the top 50 regulators and gene markers or measures of disease severity. Color denotes r value of correlation. The gene names with purple are considered as unique DEGs of psoriasis, with black are shared genes with AD. * Adjusted P < 0.05 [file Image_3.tif]
