# Supplementary material for: Finding Gene Regulatory Networks in Psoriasis: Application of a Tree-Based Machine Learning Approach
Source: Front Immunol. 2022 Jul 7;13:921408. doi: 10.3389/fimmu.2022.921408 (PMC9301015; doi:10.3389/fimmu.2022.921408)
Supplement: Supplementary file 4 [file Table_1.docx]

| **Supplementary table 1. Information of public datasets** | | |  |  |
| --- | --- | --- | --- | --- |
| **Disease** | **Publication** | **GEO ID** | **Comparison** |  |
|  |  |  |  |  |
| **Psoriasis** | Tsoi et al. 2015 | GSE63979 | 7 L vs 27 NL |  |
|  | Gudjonsson et al. 2015 | GSE67785 | 14 L vs 14 NL |  |
|  | Gudjonsson et al. 2017 | GSE83645 | 20 L vs 5 NL |  |
|  | Tsoi et al. 2019 | GSE121212 | 28 L vs 27 NL |  |
| **AD** | Suárez-Fariñas et al. 2015 | GSE65832 | 20 L vs 20 NL |  |
|  | Camille et al. 2021 | GSE140227 | 6 L vs 6 NL |  |
|  | Tsoi et al. 2019 | GSE121212 | 27 L vs 27 NL |  |
