# Supplementary material for: Finding Gene Regulatory Networks in Psoriasis: Application of a Tree-Based Machine Learning Approach
Source: Front Immunol. 2022 Jul 7;13:921408. doi: 10.3389/fimmu.2022.921408 (PMC9301015; doi:10.3389/fimmu.2022.921408)
Supplement: Supplementary file 5 [file Table_2.docx]

**Supplementary table 2. Clinical characteristics of the participants in validation cohort**

|  | **psoriasis (n=13)** | **PsA (n=15)** | **AS (n=12)** | ***P* value** |
| --- | --- | --- | --- | --- |
| **Sex** |  |  |  |  |
| Female | 7 (53.8%) | 5 (33.3%) | 3 (25.0%) | 0.302 |
| Male | 6 (46.2%) | 10 (66.7%) | 9 (75.0%) |  |
| **Age (years)** | 41.3 (13.2) | 46.3 (9.32) | 44.2 (8.54) | 0.466 |
| **BMI (kg/m^2^)** | 28.2 (7.79) | 26.9 (4.62) | 25.2 (2.66) | 0.728 |
| **Smoking** |  |  |  |  |
| never | 3 (23.1%) | 4 (26.7%) | 5 (41.7%) | 0.509 |
| past | 5 (38.5%) | 7 (46.7%) | 6 (50.0%) |  |
| present | 5 (38.5%) | 4 (26.7%) | 1 (8.3%) |  |
| **Psoriasis duration (years)** | 24.2 (14.6) | 23.1 (16.1) | - | 0.734 |
| **PASI** | 6.83 (5.40) | 5.05 (4.87) | - | 0.249 |
| **Local PASI** | 5.31 (2.56) | 5.54 (2.15) | - | 0.677 |
| **DMARD history** |  |  |  |  |
| Yes | 1 (7.7%) | 7 (46.7%) | 2 (16.7%) | 0.0433* |
| No | 12 (92.3%) | 8 (53.3%) | 10 (83.3%) |  |
| **UVB history** |  |  |  |  |
| Yes | 7 (53.8%) | 3 (20.0%) | - | 0.0623 |
| No | 6 (46.2%) | 12 (80.0%) | - |  |
| **Biologic history** |  |  |  |  |
| Yes | 12 (92.3%) | 14 (93.3%) | - | 0.916 |
| No | 1 (7.7%) | 1 (6.7%) | - |  |
| **ESR (mm/hour)** | 9.46 (12.9) | 8.27 (9.61) | 7.82 (7.78) | 0.922 |
| **CRP (mg/L)** | 4.97 (7.30) | 5.04 (6.85) | 4.94 (5.22) | 0.955 |

Mean (standard deviation) are presented for continuous values. Frequencies (proportion) are presented for categorical values. DMARD, UVB or biologic history: usage in past 3 months.

*Significant at *P* value < 0.05.
